# Designing and deploying caller tunes on mobile phones to promote malaria vaccine uptake in Africa: can the technology acceptance model (TAM) help?

**DOI:** 10.1186/s12936-024-05134-3

**Published:** 2024-11-02

**Authors:** Stanley Eneh, Francisca Onukansi, Ogechi Ikhuoria, Temitope Ojo

**Affiliations:** 1 Department of Community Health, Obafemi Awolowo University, Ile-Ife, Osun Nigeria; 2 Ivan Research Institute, University of Nigeria, Enugu, Nigeria; 3 Department of Public Health, Federal University of Technology Owerri, Owerri, Nigeria; 4 Department of Biochemistry, University of Port Harcourt, Choba, Nigeria

**Keywords:** Malaria, Malaria vaccine, Technology acceptance model (TAM), RTS,S, Caller tune

## Abstract

Malaria remains a significant global health challenge, with millions of cases and high mortality rates annually, especially in low-income countries. Africa bears a substantial burden, with direct costs of malaria among children under five reaching millions of dollars in countries like Ghana, Tanzania, and Kenya. In 2021, over 610,000 malaria-related deaths were reported, 96% of which occurred in sub-Saharan Africa. Despite existing interventions, such as long-lasting insecticidal nets, indoor residual spraying, and intermittent preventive treatment, the re-emergence of malaria underscores the need for innovative preventive strategies. This study explores the potential of utilizing mobile phone caller tunes to raise awareness and promote the uptake of the RTS,S malaria vaccine. The technology acceptance model (TAM) provides a framework for understanding how users perceive and adopt new technologies. Caller tunes, a mobile phone feature that plays audio for callers waiting to be connected, have been effective in health communication campaigns in Asia and Africa. This approach could be leveraged to enhance malaria vaccine awareness, particularly in low-income countries where vaccine hesitancy is prevalent and malaria endemic. Overall, mobile technologies have significantly improved healthcare delivery in Africa, facilitating communication, monitoring, and treatment adherence in remote areas. Integrating caller tunes with health messages about the malaria vaccine could address vaccine hesitancy and improve uptake. This would require collaboration with telecommunication companies, healthcare providers, and policymakers to design culturally and linguistically appropriate messages. However, the cost of caller tune services, the need for internet access, and cultural differences are the expected challenge that may occur in this approach. Therefore, strategic partnerships and intersectoral approaches can mitigate these issues, making caller tunes a viable tool for public health communication. Raising awareness through this innovative method could enhance the adoption of the RTS,S vaccine and support ongoing malaria control efforts in Africa**.**

## Background

Globally, malaria continues to be a leading cause of morbidity and mortality, with 400–900 million cases of illness and up to 249 million fatalities annually [1, 2]. This disease is prevalent in low-income nations [3]. The economic burden of malaria in Africa is significant. In Ghana, the average household incurs a cost of US$20.29 per malaria case. In Nigeria, annual malaria-related expenditures during pregnancy alone amount to $75.5 million. In Benin, malaria affects 1.8 million children under five each year, resulting in an estimated economic burden of $193 million (95% CI, $192–$193 million) due to treatment costs and productivity losses [4–6].

In 2021, there were over 610,000 reported deaths worldwide, with 96% occurring in sub-Saharan Africa [1]. The persistent increase in malaria cases yearly signals a potential resurgence, especially in areas with inadequate preventive measures [3]. This urgent challenge calls for innovative implementation and preventive strategies to control malaria in Africa. One promising implementation approach is using caller tunes on mobile phones to raise awareness about the uptake of the malaria vaccine (RTS, S).

Acknowledging the existing strategies and intensive campaigns used in the fight against malaria, such as Roll Back Malaria (RBM) and other related programs is imperative. The impact of RBM has significantly reduced the malaria burden since its inception [7]. Tremendous efforts and interventions, including long-lasting insecticidal nets (LLINs), indoor residual spraying, and intermittent preventive treatment for children (IPTc), infants (IPTi), and pregnant women (IPTp), have been deployed to eradicate malaria in Africa [8]. The recent malaria phase trials have demonstrated the effectiveness of the malaria vaccine (R21/RTS, S). Given the profound impact of malaria on children under five and pregnant women in Africa [8], it is crucial to drive further action and innovation in combating this disease. However, more work and advanced strategies are imperative, especially in this technological era.

The modern society operates within an advanced technological landscape, where AI and software profoundly shape daily activities and interactions [9]. The impact of digital ecosystems on human life is immeasurable, permeating various sectors, including healthcare, where they are integral to practices like e-health, m-health, and telemedicine [9, 10]. In health research, particularly clinical trials, phone-based interventions play a crucial role in medical care and addressing global health issue [11]. Leveraging these advanced technologies can help manage and combat malaria in communities. Digital communication strategies can promote the use of LLINs, IPTi, IPTp, IPTc, as well as IRS and home management of malaria through various media channels. These strategies can also strengthen vaccine uptake and address vaccine hesitancy.

Poor perception can lead to vaccine hesitancy, a growing public health challenge that significantly impacts immunization programmes in low-income countries and threatens already fragile health systems in Africa [12]. To combat malaria vaccine reluctance and encourage vaccine uptake, strategic and effective communication is essential. Considering the widespread use of mobile technology, the impact of the pandemic, and prevailing perceptions towards new vaccines, it is crucial to address the challenges in malaria immunization. Africans' acceptance of the COVID-19 vaccine was driven by the disease's virulence [13, 14]. However, in sub-Saharan Africa, the belief that malaria is not inherently dangerous poses a significant challenge (immunization gap) to the uptake of newly approved malaria vaccines in some parts of the continent.

To successfully integrate the newly approved malaria vaccine into national immunization programs in Africa, substantial efforts are needed to address population perceptions, acceptability, and beliefs. Massive awareness campaigns and innovative communication strategies are essential to overcome these challenges. Utilizing mobile caller tunes and the technology acceptance model (TAM) can effectively influence the perceptions, acceptability, and beliefs of the general population towards the malaria vaccine across African countries.

## Lessons from African contexts

In several African countries, it is common for mobile phone users to hear a song or message instead of the traditional ringing sound, known as a caller tune. A cross-sectional study conducted in Ghana applied the technology acceptance model (TAM) to explore the potential of using caller tunes as a tool to enhance medication adherence. The study's findings revealed that, consistent with TAM, individuals who perceived the application as easy to use and beneficial were more likely to intend to use caller tunes to improve medication adherence. Moreover, for those already using caller tunes, the availability of the service as a free download was associated with a stronger intention to adopt this approach. These results suggest that caller tunes could serve as an innovative and effective method for promoting medication adherence [12].

Similarly, another study utilized the technology acceptance model to examine how factors such as perceived ease of use, perceived usefulness, attitude, and cost-free availability influence the intentions of blood donors and non-blood donors to download blood donation-themed caller tunes as a means of promoting blood donation in Ghana. The study found that perceived usefulness significantly influenced the intention to use caller tunes across all groups, with the most substantial effect observed among blood donors with caller tunes (β = 0.293, P < 0.001) and non-blood donors with caller tunes (β = 0.278, P < 0.001) [15].

Despite these insights, there is a scarcity of research on this topic within the broader African context, with most studies, including the ones mentioned, being conducted in Ghana under different contextual conditions. This lack of comprehensive research highlights the need for further studies across various African settings to fully understand the potential of caller tunes in promoting health-related behaviors and other social interventions.

## Health innovative research development, cost and vaccine provision bottleneck

Technological advancements and innovative clinical investigations have led to the development of malaria vaccines, such as RTS,S, heralded as a potentially cost-effective intervention against malaria [16]. This vaccine marks a significant milestone in malaria prevention, being the first of its kind approved as an additional preventive measure to bolster existing malaria control strategies [17]. Moreover, on October 2, 2023, the World Health Organization approved a newly developed malaria vaccine called R21/Matrix-M, anticipated to complement RTS,S and enhance malaria control efforts in Africa [18]. While the initial implementation of this vaccine in Malawi, Ghana, and Kenya showed promise [19], challenges remain, particularly regarding cost and governmental readiness to provide vaccines for children. For instance, the estimated cost of administering a three-dose regimen alongside a booster shot stands at around $4 [8, 10], presenting a formidable challenge for countries such as Nigeria struggling with vaccine provision and distribution.

## Digital opportunities and mobile innovations in health

The increasing mobile subscription rates in Africa present a significant opportunity to utilize mobile phones in addressing healthcare challenges [10]. Mobile innovations including caller tunes have opportunities to strengthen service delivery. Healthcare providers should be willing to integrate caller tunes into their mobile devices to assist individuals struggling with medication adherence. Conducting comprehensive research to determine the impact of caller tunes on vaccine/medication adherence is crucial. Ideally, this research should be grounded in a theoretical framework known for its effectiveness in promoting medication adherence [20].

Mobile technologies have been deployed, tested, and seem to be effective in health care delivery, and mobile health (mHealth) tools have significantly helped African health workers and researchers provide clinical services in remote places, hard-to-reach communities, and outside the clinical setting [21]. The current innovative approach brought by technology makes health care services accessible to patients and reduces travel expenses. Furthermore, mobile devices have been instrumental in supporting community case management for malaria in various communities across Kenya and other parts of Africa. They facilitate improved communication and monitoring of primary healthcare centres and serve as reminders and alerts for patients to adhere to their malaria medication [19, 22, 23]. Additionally, mobile devices have been utilized to monitor and enhance diagnostic services provided by community health workers in remote areas [22]. Mobile technology enables health workers to monitor patients at home and manage malaria treatment through SMS reminders [21]. Given its significant role in medical services, especially during the pandemic [22], mobile technology can be leveraged to assess perception and acceptability and create massive awareness of the newly approved malaria vaccines.

## Case example: application of technology acceptance model (TAM) in designing and deploying caller tunes on mobile phones for malaria to promote malaria vaccine uptake in Africa

**Fig. 1:**
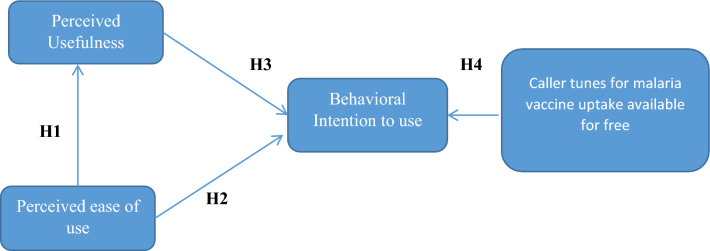
Technology Acceptance Model (TAM) Hypotheses. H1: Perceived ease of use is positively related to perceived usefulness of caller tunes. H2: The intention of using caller tunes for malaria vaccine uptake awareness is strongly correlated with the perceived ease of use. H3: Perceived usefulness is positively related to intention to use caller tunes. H4: Free caller tunes positively associated with intention to use

The TAM has been widely recognized since 1989 as a valuable tool for determining user perceptions and acceptance of technology within the general population. The TAM emphasizes that perceived usefulness and ease of use, both influence the adoption of technology. Research has shown that perceived usefulness enhances the perception of ease of use, which in turn influences the intention to use the technology, ultimately determining its actual adoption [12]. Given the potential benefits of the TAM framework in designing and deploying caller tunes on mobile phones to promote malaria vaccination in Africa, it is essential to consider the framework’s constructs (see Fig. 1) to guide the design of caller tunes on mobile phones and future pilot studies aimed at generating evidence-based results.

The following constructs of the technology acceptance model (TAM) will be explored in the context of this study, which aims to promote malaria vaccine uptake across Africa through caller tunes on mobile phones.

## Perceived usefulness

Perceived Usefulness: refers to how users evaluate the benefits and advantages of a particular application, particularly in terms of its practicality, efficiency, and ability to meet their needs. It plays a pivotal role in influencing whether individuals adopt and continue using the application. For example, a study from Ghana demonstrated that users' perceptions positively influenced medication adherence [12]. Similarly, in the context of construction firms, users' perceived usefulness significantly impacted the adoption of AI-based technologies [24]. Additionally, perceived usefulness has been shown to encourage reporting of adverse drug reactions among patients [10]. The belief that a given technology is useful can shape attitudes toward its use and enhance the intention to adopt it, as observed in voluntary blood donation [15]. In the current study, the proposed hypotheses are outlined in Table 1 (H1).

**Table 1:** The Technology Acceptance Model (TAM) hypotheses and constructs

TAM constructs/hypotheses		
Perceived Usefulness of application	H1: Perceived ease of use is positively related to perceived usefulness of caller tunes	Perception that caller tunes would be a practical technological tool for encouraging the adoption of the malaria vaccination
Behavioral Intention to use	H2: The intention of using caller tunes for malaria vaccine uptake awareness is strongly correlated with the perceived ease of use	Aim to improve malaria vaccine adoption by using caller tunes on mobile phones
Perceived ease of use	H3: Perceived usefulness is positively related to intention to use caller tunes	How many persons believe it is simple to subscribe to caller tunes on their mobile phones to advertise the malaria vaccine
Free of Cost	H4:Free caller tunes positively associated with intention to use	To encourage the use of the malaria vaccination, providing caller tunes for free to individuals who wish to subscribe

## Intention to use

Behavioural intention to use refers to an individual's favourable or unfavourable feelings toward performing the target behaviour. A study in Turkey on mobile health adoption found that an individual's intention to use mobile health services was significantly influenced by their behaviour [25], which is similarly to other findings from Ethiopia [26]. For the proposed hypothesis, See Table 1 (H2) for more information.

## Perceived ease of use

The extent to which an individual perceives a specific technology will be simple and easy to use [27]. A study conducted in China suggests that perceived ease of use significantly influences perceived usefulness [28]. Similarly, studies on the intention to use mhealth services in Ethiopia, Taiwan, and Malaysia, respectively[27, 29, 30] have demonstrated that perceived ease of use directly and positively impacts users' intention to use mobile phone services, as well as their perceived usefulness and attitudes toward these technologies. However, the proposed hypotheses are outlined in Table 1 (H3).

## Free of cost

Intention to use without financial cost: studies have revealed a significant association with the intention to use the application [12, 31]. Furthermore, a study from Jordan [31] indicated that cost did not affect the intention to use this technology, while another study found that cost did influence the acceptance of mobile technology [32]. For the proposed hypothesis, see Table 1 (H4) for more information.

Therefore, given this belief that a given technology, TAM is useful and can shape attitudes to address perceptions, acceptability, and beliefs regarding malaria vaccines among Africans using caller tunes as a promotional tool could prove highly beneficial in promoting malaria vaccine uptake in Africa.

Caller tunes, a mobile phone feature that plays music, voicemails, or other sound effects for callers while they wait for the recipient to answer, remains an underutilized resource for promoting awareness and communication strategies, particularly for vaccine uptake [16]. While mobile phone technology has been extensively deployed for health services through alerts, SMS text-based interventions, and medication adherence, caller tunes have not been effectively harnessed for this purpose [17]. Caller tune services are prevalent and widely used in Asia and African countries, serving as an effective medium for communication strategies. In India, for example, celebrity voices were utilized in 2013 to raise awareness about various health issues, such as cervical cancer, obesity, stress, cardiovascular diseases, and breast cancer [16].

However, using the technology acceptance model (TAM) will offer valuable insights into African perceptions and beliefs regarding the newly approved malaria vaccine which would guide potential future pilot studies (See Fig. 1 and Table 1). Baseline data from the potential pilot study will enable national health officials to tailor strategic communication interventions aimed at alleviating fears and promoting vaccine uptake upon availability (See Table 2). Furthermore, collaboration with telecommunications companies to deploy caller tunes as a means of communicating about malaria vaccine uptake is essential, given that all mobile service providers offer this service to enhance customer experience. By adopting TAM for malaria vaccine acceptance, there is potential for enhanced inter-sectoral collaboration with telecommunication companies, aligning with the one health approach and to support collaborative fight against malaria in Africa (See Table 3).

**Table 2:** Partnerships and Inter-sectoral approaches toward malaria vaccine Uptake caller tune creation: how it might function

Strategies	Factors to be considered
*Songs*: 1) Involving African popular musicians to formulate and produce songs for malaria vaccine uptake, which would be used as a caller tunes by different telecommunication companies 2) In creating the songs for Malaria vaccine, local languages should be considered to properly communicate with illiterate or uneducated populations	1) Consider to involve celebrity that are well known for promoting social and well being of the masses 2) Health and other concerned professional bodies must ensure to regulate the songs and audio messages formulated for malaria vaccine uptake to avoid passing false information
*Messages, Audio, and Adverts: involving* African movie industries, African celebrities and influential personality such religious leaders, footballers, comedians	3) The formulated songs and messages must be regulated to reduce fear among masses

**Table 3:** Some of the telecommunication companies in Africa with their mobile caller tunes strategies

Country	Telecommunication company	Mobile caller tune information
Nigeria	MTN	‘‘With the help of MTN Callertunez, you may replace the uninteresting "ring-ring" tone people use to phone you with a custom melody of your preference’’. https://www.mtn.ng/play/callertunez/
Nigeria	Globacom	‘‘Every time your phone rings, the Glo Caller Tunes service provides a variety of local and foreign music to keep your callers entertained while giving you a distinctive appearance’’. https://www.gloworld.com/ng/glo-caller-tunes
Nigeria	Airtel	‘‘Every call becomes rhythmic when you use the Airtel Hello Tunes; you have the option to substitute the default music with a broad selection of Tunes that your callers will enjoy’’. https://www.airtel.com.ng/vas/music/hellotunes
Ghana	Vodafone	‘‘Before you answer your callers, entertain them with awesome tunez. While customers wait for their calls to be answered, you can assign as many songs for your callers to listen to’’. https://vodafone.com.gh/personal/mobile/vas/caller-tunez/
Uganda	Uganda Telecom	‘‘Get rid of the monotonous "ring, ring" tone that appears when people phone you. Utilize UTL Caller Tunes to control what callers hear when they dial your number’’. https://www.utl.co.ug/personal/value-added-services/caller-tunes/
Kenya	Safaricom	‘‘You can now provide your callers with entertainment by playing their favorite SKIZA song for just Ksh 1.5 per day for both local and foreign tunes. https://www.safaricom.co.ke/personal/value-added-services/entertainment/skiza-tunes
Mauritius	Emtel	You may now persuade your friends to play their favorite song whenever they call you. Play well-known music for as many of your pals as you like. Choose your tune from wacky sounds to pure instrumentals, international hits to Bollywood hits, and much more. You can either choose a single tune for everyone or create a tune specifically for each caller’’. https://www.emtel.com/services/value-added-services/caller-tunes
Liberia	Lonestar Cell MTN	‘‘Using the Lonestar Cell MTN Caller Tunes service, you can use your favorite tune as a ringback tone to amuse callers’’https://lonestarcell.com/caller-tunez/

### Expected and possible limitation of this approach and strategic recommendation

Notwithstanding, employing caller tunes as a health awareness tool would require several prerequisites to harness its potential. Telecommunication companies would need to permit the downloading and utilization of caller tunes on their networks. Satisfying all the aforementioned prerequisites would require time, adequate internet, cost and several inter-sectoral collaborations.

*Cost and free caller tunes activation*: the monthly cost of caller tunes from telecommunication companies could be a challenge in implementing these approaches. There is a possibility that Africans will not be able to afford the cost of the services provided, which would result in disabling the caller's tuning strategies. The monthly cost of caller tunes from telecommunication companies may present a challenge in implementing these strategies. The affordability of these services in Africa could impact the effectiveness of caller tune campaigns, potentially leading to their discontinuation. However, if telecommunications providers in Africa are willing to offer malaria vaccine caller tunes at no cost, it could significantly enhance awareness of the malaria vaccine, reduce vaccine hesitancy, and promote One Health approaches across the continent.

*Mismatching of caller tunes*: Africa is a continent of diverse cultures, beliefs, languages, traditions, and religions. Given that telecommunications firms have the capability to reach extensive geographical regions encompassing numerous distinct communities, the variations in cultural backgrounds, beliefs, and religions may mean that not all caller tunes will be universally well-received within the same country. Therefore, a one-size-fits-all malaria vaccine uptake message may not be effective for everyone, even within the same country. It is crucial to consider local norms, traditions, languages, and cultures when designing malaria vaccine uptake strategies across African communities. Caller tunes should be customizable, allowing users to select options based on their preferences, such as religion, belief, or culture. This approach will help avoid mismatches and promote effective communication strategies for malaria vaccine uptake across Africa.

*Internet access*: utilizing caller tunes may result in the exclusion of many individuals from lower socioeconomic backgrounds, specific geographical areas, those with lower educational attainment, illiterate individuals, and those lacking internet access. Which must be considered to enhance proper malaria vaccine awareness and uptake.

*Partnerships and inter-sectoral approaches toward malaria vaccine uptake, caller tune creation and its potential use*: an intersectoral approach may comprise numerous departments, government bodies, non-governmental organizations, key players, and other parties with the aim of resolving a specific issue. Current comparative studies from Ghana and India [13, 14] have highlighted the significant positive impact and importance of intersectoral collaboration in supporting One Health policy. Therefore, it will be a striking strategy to involve telecommunication companies that offer different caller tunes, medical practitioners, health agencies, African celebrities, social media influencers, comedians, musicians, in formulating and creating malaria vaccine uptake songs and messages.

Telecommunication companies across Africa are known with different caller tunes and call back techniques (See Table 3) and should incline to offer free caller music downloads and subscriptions as a component of their corporate social responsibility initiatives aimed at advancing public health [12]. Establishing strategic partnerships with telecommunication companies can significantly alleviate or completely cover the expenses tied to caller tunes, thereby enhancing extensive awareness efforts.

In addition, massive awareness regarding RTS, S vaccine uptake in Africa is essential, as Nigeria and others African countries such as Benin, Burkina Faso, Burundi, Burundi, Cameroon, Democratic Republic of the Congo, Liberia, Niger, Sierra Leone, and Uganda are expected to receive the vaccine in their home countries, which can be incorporated into African health routine immunization programs for vaccine uptake sustainability. In creating malaria vaccine caller tunes across African countries, it will be wise to consider religion and languages, which will directly and indirectly influence vaccine uptake (See Table 1). Although for a successful use of caller tunes as an approach to raising malaria vaccine uptake in Africa, a lot of conditions need to be considered by employing TAM and conducting a pilot study.

## Conclusion

The ongoing global battle against malaria, particularly in sub-Saharan Africa, underscores the urgent need for innovative strategies to combat this pervasive disease. Despite significant efforts through initiatives like Roll Back Malaria (RBM) and the development of vaccines, such as RTS,S, and R21/Matrix-M, malaria remains a significant economic and public health burden. The widespread adoption of advanced digital communication technologies, including mobile caller tunes, presents a promising avenue to enhance malaria vaccine awareness and uptake. However, successful implementation requires careful consideration of the socio-cultural and economic diversity within African countries. Strategic partnerships with telecommunications companies, along with tailored, culturally sensitive messaging, are essential to overcoming challenges such as cost, internet access, and varying local perceptions.

Additionally, the limited research on the use of caller tunes for health promotion within the broader African context—where most existing studies have been conducted in Ghana under different conditions—highlights a critical gap in the literature. To fully harness the potential of caller tunes in promoting malaria vaccine uptake, further studies are needed across various African settings. These studies should focus on understanding the specific socio-cultural dynamics and technological infrastructure that could influence the effectiveness of such interventions. By integrating these approaches with existing health initiatives and utilizing frameworks like the technology acceptance model (TAM), which can significantly reduce malaria-related morbidity and mortality across the African continent.

## Data Availability

No datasets were generated or analysed during the current study.

## Abbreviations

TAM: Technology acceptance model
RBM: Roll Back Malaria
AI: Artificial Intelligence
LLINs: Long-lasting insecticidal nets
IPTc: Intermittent preventive treatment of malaria in children
IPTi: Intermittent preventive treatment of malaria in infant
IPTp: Intermittent preventive treatment of malaria in pregnant women
